# Roles of Hepatitis B Virus Mutations in the Viral Reactivation after Immunosuppression Therapies

**DOI:** 10.3390/v11050457

**Published:** 2019-05-19

**Authors:** Jun Inoue, Takuya Nakamura, Atsushi Masamune

**Affiliations:** Division of Gastroenterology, Tohoku University Graduate School of Medicine, Sendai 980-8574, Japan; ikuyokuruyo888@gmail.com (T.N.); amasamune@med.tohoku.ac.jp (A.M.)

**Keywords:** HBV, reactivation, immune escape mutation, HBsAg

## Abstract

Reactivation of hepatitis B virus (HBV) is a major problem in patients receiving chemotherapy for malignant diseases or immunosuppression therapies. It has been thought that a reduction in the immune responses might result in the reactivation of HBV replication from covalently closed circular DNA (cccDNA) residing in hepatocytes. However, not only the host’s immune status, but also viral mutations have been reported to be associated with reactivation. Especially, several case reports about amino acid mutations in hepatitis B surface antigen (HBsAg) that escape from immune reactions have been reported, and recent reports showed that the frequencies of such mutations are higher than previously expected. In this review, we summarize the characteristics of viral mutations, including immune escape mutations in HBV-reactivated patients, and discuss their significance.

## 1. Introduction

Hepatitis B virus (HBV) infection is a worldwide health problem. HBsAg-positive patients with high levels of serum HBV DNA and alanine aminotransferase (ALT) are targets of anti-viral therapies because they are at high risk of liver cirrhosis and hepatocellular carcinoma (HCC) [1]. It is estimated that almost one third of people in the world experience infection [2] and, among them, almost 248 million persons are estimated to have hepatitis B surface antigen (HBsAg) [3]. Generally, HBsAg-positive persons are considered to have HBV persistent infection, and those in whom HBsAg has cleared are thought to be in a cured status. It is known that anti-cancer chemotherapies or immunosuppressive therapies can induce HBV reactivation not only in HBsAg-positive patients, but also in HBsAg-cleared patients [4]. What is the cause of HBV reactivation even in such patients?

In HBV infection, T-cell and B-cell responses affect the outcome of the liver disease [5]. These responses are essential to control HBV infection in the natural course. HBV-specific T-cell responses suppress viral replication by both cytopathic effects [6] and non-cytopathic cytokine pathways [7]. B-cells produce neutralizing antibodies against HBV and inhibit the spread of HBV infection to other hepatocytes. It has been revealed that covalently closed circular DNA (cccDNA) persists in the hepatocytes of patients who cleared HBsAg with such immune responses [8]. The presence of cccDNA and the weakened immune responses are thought to enable HBV reactivation in patients with resolved infection [9]. Because HBsAg-cleared patients have antibodies against HBs (HBsAb) and/or those against hepatitis B core (HBcAb), patients with these antibodies should be monitored carefully during chemo/immunosuppression therapies [9].

The definition of HBV reactivation was proposed by the American Association of the Study for the Liver Diseases in 2013 as either an exacerbation of chronic HBV infection or reactivation of past HBV infection after the start of immunosuppressive therapy [10]. An exacerbation of chronic HBV infection was defined as HBsAg positivity with ≥2 log10 increase in the HBV DNA levels from the baseline levels, an HBV DNA level of >100 IU/mL in a person with undetectable HBV DNA at baseline, or an HBV DNA level of ≥100,000 IU/mL in a person whose HBV DNA was not tested at baseline. Reactivation of past HBV was defined as reverse HBsAg seroconversion (HBsAg-negative becomes HBsAg-positive), or the appearance of HBV DNA in the absence of HBsAg. 

In regard to the factors associated with HBV reactivation, not only the types of drugs used for chemo/immunosuppression therapies, but also the viral mutations have been reported. Recently, the high frequency of HBsAg mutations in HBV-reactivated patients has been reported [11,12]. HBV with these HBsAg mutations is considered to escape from recognition by the immune system, and this may be important in considering the mechanisms of the HBV reactivation. Here we summarize the recent findings on the viral mutations, including immune escape mutations found in HBV-reactivated patients with chemo/immunosuppression therapies, and we discuss the mechanisms of HBV reactivation.

## 2. HBsAg Mutations Found in HBV-Reactivated Patients After Chemotherapies/Immunosuppression Therapies

The envelope proteins of HBV are encoded by the preS/S gene. Three hepatitis B surface proteins (large (LHBs), middle (MHBs), and small (SHBs or HBsAg)) are translated from three different initiation codons to the common termination codon. The amino acid sequence of HBsAg consists of three major parts: N-terminal region (amino acids 1–99), major hydrophilic region (MHR, amino acids 100–169), and C-terminal region (amino acids 170–226). MHR contains major epitopes exposed on the surface of HBV particles, and especially, the “a” determinant (amino acids 124–147) is a major target of neutralizing antibodies [13,14].

The escape mutations in the “a” determinant were reported to be present in HBV-infected patients who had been vaccinated [13]. It is known that the binding affinity of HBsAb to HBsAg with these mutations is weak. An HBV-reactivated case after chemotherapy who had an escape mutation was reported first by Carman et al. in 1995 [15]. The case developed fulminant hepatitis and had the G145R mutation in the “a” determinant. Subsequently, some case reports showing HBV-reactivated patients with escape mutations in HBsAg appeared [16,17]. Recently, Salpini et al. reported that 79% of 29 HBV-reactivated patients with immunosuppression therapy had HBV with amino acid mutations in the immune-active HBs regions based on results from direct and ultradeep sequencing using plasma samples [12]. All patients were infected with genotype D HBV, and the percentage was significantly higher than in patients with chronic infection of genotype D HBV. Most (8/13) of the mutations are located within MHR, including the “a” determinant, and, of note, some (5/13) mutations were found in class I/II-restricted T-cell epitopes, suggesting that escape from T-cell responses might play a role in HBV reactivation. The mutations in MHR contain those making additional N-linked glycosylation (NLG) sites, which reduce HBsAb recognition. Colson et al. performed direct sequencing of S gene using serum samples from 16 HBV-reactivated patients who were negative for HBsAg before chemotherapy, and showed that all patients had at least one amino acid mutation in MHR [11]. Most patients were genotype D (13/16), and it is considered that escape mutations in MHR of HBsAg might be associated with most HBV reactivations after immunosuppressive therapies, at least in patients with genotype D HBV. In Table 1, the mutations in HBsAg that were found in HBV-reactivated patients are summarized. Most mutations were identified with direct sequencing of S gene using serum samples. As shown in the table, some mutations were reported to reduce the recognition by antibodies using in vitro assays such as the western blotting analysis and enzyme-linked immunosorbent assay (ELISA). It should be taken into account that the wild type amino acids at some positions differ among genotypes. Additionally, several mutations could induce the overlapping transcriptase domain of polymerase, including rtA181T/S, which is known to cause drug resistance. Although these polymerase mutations might affect the viral replication, their significance in the reactivation is unknown. To elucidate the general roles of HBsAg mutations in HBV reactivation, more HBV-reactivated patients, including those with HBV of genotypes other than genotype D, have to be analyzed in future studies.

## 3. Mechanisms by Which Immune Escape Mutations Arise in HBV-Reactivated Patients

As described above, immune escape mutations in HBsAg were found in patients with HBV reactivation, although the frequencies could vary among the genotypes of HBV. Why are such mutations present? A possible reason is the selection of immune escape mutants during the treatment with immunosuppressive therapies. In HBsAg-disappeared patients, the immune system, including HBsAb in the serum, suppresses HBV replication. HBsAb in excess binds to HBsAg and inhibits the detection of low-level HBsAg in most assays. Also, it binds to the infectious HBV particles (Dane particles) and inhibits the entry to hepatocytes. Even if a small amount of immune escape mutant emerges as a random event, excessive HBsAb might inhibit the spread of mutant HBV. In an immunosuppression state, the production of HBsAb decreases, and Dane particles with immune escape mutations, which are less recognized by HBsAb, might become easier to enter into uninfected hepatocytes, which might spread the infection. Therefore, less or an undetectable level of HBsAb at the start of immunosuppressive therapy could be a risk factor for HBV reactivation [42,43,44,45]. In this context, the escape mutations might be results from reduced selection pressure, and their high frequencies in reactivated patients might indicate that these have critical roles during the reactivation. Additionally, the administration of glucocorticoids, which stimulate glucocorticoid-responsive element (GRE), increases the transcription from cccDNA [46] and might boost the chance of mutation.

Another possible reason is underestimation of the HBsAg level because of immune escape mutations. As shown in Table 1, many mutations in MHR were reported to reduce recognition by antibodies and might reduce the signal of HBsAg detection with various methods such as ELISA and chemiluminescent immunoassay (CLIA). To detect immune escape mutants, some methods use a polyclonal antibody or a mixture of some monoclonal antibodies, but there is still a possibility that HBsAg signals are reduced by the presence of such mutations [47]. Therefore, an HBV carrier with escape mutants can be misdiagnosed as in a state of resolved infection because of the presence of HBsAb and/or HBcAb. Being unaware of HBV infection might lead to HBV reactivation from the lack of prophylaxis with the nucleos(t)ide analogue. We should pay an attention to such patients, including occult infection of HBV.

## 4. Precore Mutation in Patients With HBV Reactivation

Several papers showed that the precore G1896A mutation, which makes a stop codon in the precore/core protein and abrogates the HBeAg expression, was associated with HBV reactivation [48,49,50,51,52]. Fatal cases of HBV reactivation harboring this mutation have been reported [53,54]. Also, some papers reported that the core promoter mutations of A1762T/G1764A were found in the reactivated patients. The association between these mutations and fulminant hepatitis in acute infection has been reported [55]. The precore mutation is considered to make the replication capacity higher if there are no or only weak adaptive immune responses [56]. On the other hand, these mutations were found frequently in the inactive HBV carriers, indicating that the HBV clones with these mutations are selected after pressure from the immune responses. These mutations reduce the expression of HBeAg, which is considered to have an immunoregulatory role and to be required for the development of persistent infection [57]. Therefore, HBV with the core promoter/precore mutations has high replication capacity and can cause stronger immune responses. If these mutations are present with immune escape mutations, it might enhance the HBV reactivation further. Although the frequency of the co-presence of these mutations has not been reported, we previously reported a genotype C HBV-reactivated case with immune escape mutations of P120A + G145R and mixed type precore mutation (G1896R) [23].

## 5. Conclusions

Immune escape mutations in HBsAg were frequently found in HBV-reactivated patients. The frequency was reported mainly in patients with genotype D HBV, but further studies in patients with HBV of genotypes other than D will be required to reveal the difference among HBV genotypes. The emergence of mutants might suggest the mechanisms of HBV reactivation, which could be important for the prevention of reactivation.

## Figures and Tables

**Table 1:** Reported mutations of HBsAg that were found in HBV-reactivated patients with chemotherapies/immunosuppression therapies.

HBsAg Region	Amino Acid Position	Specific HBV Genotype ^a^	Mutant Amino Acid	References	Note ^b^
N-terminal	F8	A, C, D	L, P	[11]	*rtI16T, rtP17T/A*
	C48		G	[12]	*rtV56G*
	V96		A	[12]	
MHR	Y100			[12]	Reduces recognition by antibodies in vitro (Y100S) [18]
	M103		I	[11,12]	*rtV112F/L/I*
	L109		Del, I, Q	[11,12]	*rtS117Y*
	L110	C	R, I	[16,19]	Shown only in case reports, *rtT118N*
	S114	B, C, D		[12]	
	T115			[12]	
	T116		N	[11]	Reduces recognition by antibodies in vitro [18], make an additional N-glycosylation site [20], *rtH124Q*
	T118		K	[12,21]	Reduces recognition by antibodies with P120Q in vitro [22], *rtH126Q*
	P120		A, T	[12,23,24]	Reduces recognition by antibodies in vitro (P120A) [25], *rtT128S/N*
	R122	D	K	[16]	Reduces recognition by antibodies in vitro [26], *rtP130Q*
	K122	A, B, C	R	[24,27]	Reduces recognition by antibodies in vitro [27]
^c^	C124		N	[17]	Shown only in a case report, *rtL132K*
^c^	T126	A, B, D	N, I	[11]	*rtD134E*
^c^	I126	C	T	[11]	
^c^	P127		S	[28]	Shown only in a case report, *rtS135F*
^c^	Q129		R	[28]	Shown only in a case report
^c^	G130		R	[17]	Reduces recognition by antibodies with M133T and F134L in vitro [29], *rtR138K/T*
^c^	M133		T	[24]	Makes an additional N-glycosylation site [30]
^c^	F134	A, B, C	I, Y, S, L	[11,16,24]	Reduces recognition by antibodies in vitro (F134S) [31], *rtV142D, S143T/A*
^c^	Y134	D	F, N, H	[11,12]	*rtV142E/A*
^c^	S136		Y, F	[11]	Reduces recognition by antibodies in vitro (S136Y) [32]
^c^	P142		L	[16]	Reduces recognition by antibodies in vitro [33]
^c^	S143	C, D	L	[12]	Reduces recognition by antibodies with Y100S or T116N in vitro [18]
^c^	D144		A, E	[11,12,16,24,27,34,35]	Reduces recognition by antibodies in vitro (D144A [36], D144E [27,37]), *rtR153G*
^c^	G145		R, A, E	[11,15,23,24,35,38]	Reduces recognition by antibodies in vitro (G145R [31], G145A [39]), *rtR153Q/P*
^c^	N146		S	[17]	Shown only in a case report
	E164		G, V	[11]	
C-terminal	S171		F	[12,17]	
	W172		L, C	[11]	*rtA181T/S* [40,41]
	S174		N	[11]	
	L175		S	[11,12,17]	
	V177		A, L	[11]	*rtS185T*
	G185		E	[12,17]	
	V190		A	[12]	
	S193		L	[11]	

MHR, major hydrophilic region. ^a^ If the wild type amino acid at each position is different among genotypes A–D, the specific genotypes with the indicated wild type amino acid are shown. ^b^ The amino acid mutations that could be caused in the overlapping reverse transcriptase domain of polymerase by the same nucleotide mutations are shown in italic type. ^c^ Amino acid positions within the “a” determinant.
